# Recent Advances in Effector Research of *Magnaporthe oryzae*

**DOI:** 10.3390/biom13111650

**Published:** 2023-11-14

**Authors:** Yun-Yun Wei, Shuang Liang, Xue-Ming Zhu, Xiao-Hong Liu, Fu-Cheng Lin

**Affiliations:** 1 College of Biology and Environmental Engineering, Zhejiang Shuren University, Hangzhou 310015, China; 12016088@zju.edu.cn; 2 State Key Laboratory for Managing Biotic and Chemical Treats to the Quality and Safety of Agro-Products, Institute of Plant Protection and Microbiology, Zhejiang Academy of Agricultural Sciences, Hangzhou 310021, China; liangs@zaas.ac.cn (S.L.); zhuxm@zaas.ac.cn (X.-M.Z.); 3 Laboratory of Rice Biology, Institute of Biotechnology, Zhejiang University, Hangzhou 310058, China

**Keywords:** *M. oryzae*, effectors, classification, function, secretion, regulation mechanism

## Abstract

Recalcitrant rice blast disease is caused by *Magnaporthe oryzae*, which has a significant negative economic reverberation on crop productivity. In order to induce the disease onto the host, *M. oryzae* positively generates many types of small secreted proteins, here named as effectors, to manipulate the host cell for the purpose of stimulating pathogenic infection. In *M. oryzae*, by engaging with specific receptors on the cell surface, effectors activate signaling channels which control an array of cellular activities, such as proliferation, differentiation and apoptosis. The most recent research on effector identification, classification, function, secretion, and control mechanism has been compiled in this review. In addition, the article also discusses directions and challenges for future research into an effector in *M. oryzae*.

## 1. Introduction

*Magnaporthe oryzae*, a hemibiotrophic ascomycete, results in the destructive blast disease of rice, wheat, and other agricultural products, which has severe economic impact [1,2]. About 6% of the entire global rice crop is thought to be destroyed annually by rice blasts, with outbreaks regularly causing up to 30% loss [3,4]. *M. oryzae* has a remarkable capacity for environmental adaptation, as evidenced by the diversity of *M. oryzae* isolates from various geographic locations [5]. *M. oryzae* needs to experience a series of developmental changes for it to flourish. These modifications allow it to interact with the surface of the crop, invade the host’s cuticle, extend inside the rice cells, and ultimately finish the pathogenic cycle.

**Figure 1:**
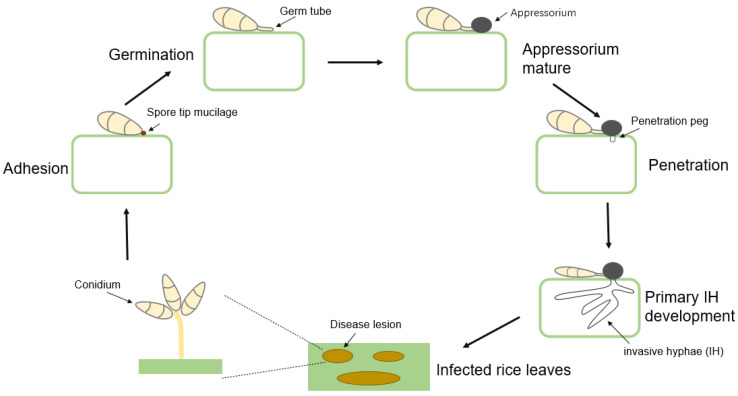
The infection cycle in *M. oryzae*.

The infection of *M. oryzae* is a complicated and specific procedure (Figure 1). The conidium that releases mucilage and adheres to the surface of the leaves is the classic sign that *M. oryzae* infection has started [6]. After the germination of conidia, the top of the germ tube further divides and expands to form a dome-shaped cell, that is identified as a unique invasive appressorium structure [7]. The appressorium continuously accumulates melanin and glycerol, and when a certain amount is reached [8] it forms an expansion pressure of up to 8.0 MPa, providing sufficient mechanical force to damage the stratum corneum of leaves of the target crop [9]. The appressorium further forms invasive pegs that penetrate the host plant epidermis and enter host cells; the differentiated invasive mycelium (IH) absorbs host nutrients and continues to expand within host tissues until *M. oryzae* adopts the necrotrophy lifestyle, which murders the alive cell and induces necrotic lesions on the host [10,11].

Active secretion of a variety of effectors by *M. oryzae* entering the host cell once the appressorium has matured is a crucial requirement for the establishment of a successful infection. These effectors are a class of small secreted proteins that play the roles of inhibitors/inducers of the basic immune response of plant cells by targeting host receptors or defense-signaling components. Although the effectors are typically low-molecular-weight proteins, they are unique in the way they manipulate the host’s machinery. In order to create the functional and structural alterations in plants’ defense, effector molecules either promote infection by the pathogen via release of virulence factors and toxins, or stimulate immunity reaction upon recognition of avirulence factors and elicitors, or both. In plant pathogenic fungi, the biggest class of effectors are functional proteins, and they are not only a key tool for plant pathogenic bacteria to successfully invade the host, but also a target for the plant immune system to recognize pathogen invasion.

In order to activate the signaling channels that regulate diverse biological events like apoptosis, proliferation, differentiation, and death, effectors interact with distinctive receptors on the surface of cells. Discovering what role *M. oryzae* effectors play and how they work has advanced significantly in the last decade. The pathogenicity of t *M. oryzae* is significantly influenced by effectors, which manipulate host plant defenses and promote infection. Researchers have concentrated on finding and characterizing these effectors with the goal of gaining an improved comprehension of the molecular mechanisms underpinning the pathogen–host relationship. Genomics, transcriptomics, proteomics and functional analysis have identified numerous effectors and revealed their diverse functions, including suppressing host immune reactions, manipulating plant metabolism and facilitating nutrient acquisition. Further studies aim to elucidate the repertoire of effectors, to understand their mode of action and utilize the knowledge gained to develop effective strategies for disease management of *M. oryzae*. The purpose of this review is to offer a complete overview of recent developments in the biology of effectors and the molecular processes by which they influence plant responses, as well as a summary of all known effectors of *M. oryzae* and their roles to date.

### Immune System in Rice–M. oryzae Interaction

**Figure 2:**
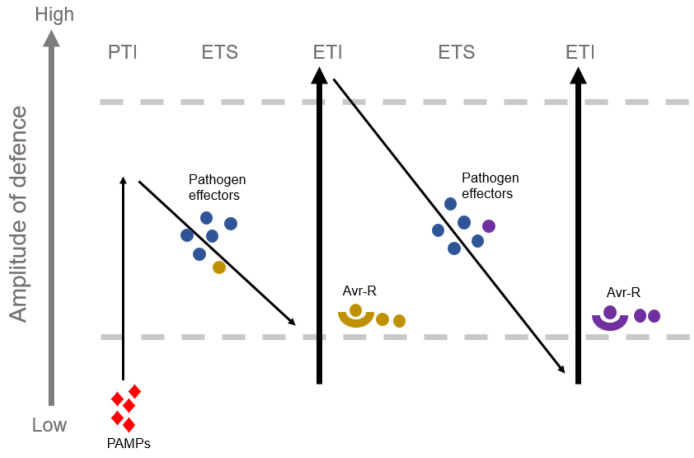
The interaction model plant and pathogen.

Plants have their own immune mechanisms (Figure 2). When pathogens attempt to enter plants, they launch plenty of immunity reactions. There are three phases of a plant’s natural immune system which can be classified, according to the location of an infection. The cuticle, cell wall, antibacterial compounds and defensive enzymes on the surface of plant cells form the first natural physical barrier [12]. Once the pathogen enters the host plant through stomata, water holes or mechanical damage, the second layer of the defensive reaction, PTI (pattern-triggered immunity), is initiated when the pattern recognition receptor on the host cell membrane discerns the conserved PAMPs (pathogen-associated molecular patterns) of the pathogenic bacteria [13]. PRR (Pattern-recognition receptor) distribution on the membrane of the cell is what causes PTI to be activated by microbial patterns [14]. PTI effectively prevents pathogen invasion [15] and maintains endophytic microbiota equilibrium in the leaf of the plant [16]. To evade or inhibit PTI, effectors of pathogen were induced and transported to host cells, leading to ETS (effector-triggered sensitivity) and interfering with defensive responses. In the stage of ETS, the effector interacts with host defensive regulators, like Slp1, and subverts PTI by competing with CEBiP (chitin elicitor binding protein) for chitin binding, which is necessary for chitin-mediated immunity in rice [17]. A similar mechanism has been demonstrated in a new paper, where it was shown that in order to suppresses PTI, chitinase1 (MoChia1) prevents the interaction with the host tetratricopeptide repeat protein (*OsTPR1*), thereby not permitting the accumulation of free chitin and discouraging the immune response [18]. Recently, it was discovered that effectors like *MoHTR1* and *MoHTR2* might be used to directly target the rice nucleus and corrupt the PTI [19]. Additionally, the third layer of immunity, referred to as effector-triggered immunity (ETI), is activated when the invasion of an effector is detected. ETI, which often results in a hypersensitive response (HR) causing cell death at the location of penetration, corresponds to escalated and exaggerated PTI [14]. A particular feature of ETI is HR, which causes fast demise of cells at the position of the pathogen invasion and inhibits the propagation of the plant disease [20].

## 2. Resistance Genes in Plant

The activation and expression of the *R* gene is one of the defense measures of the vegetation’s immunological program. Most of these *R* genes manipulate networks of downstream general defense pathways, which are primarily involved in the immune system reactions they control [21]. One of the greatest affordable, efficient, and environmentally friendly alternatives for supervising *M. oryzae* is a combination of *R* genes for resistance [22]. However, the *R* gene found in rice varieties is often defeated after release because pathogens continuously evolve virulent strains targeting specific *R* genes [23]. To effectively manage blast in rice production, it is crucial to isolate novel genes which exhibit a broad range of defense against *M. oryzae* from diverse rice cultivars. Over the past few decades, comprehensive genetic and linkage examinations have identified and mapped more than a hundred blast *R* genes across various rice chromosomal regions, and thirty-eight of these have been successfully cloned and characterized [24] (Table 1). Approximately sixty percent of the *R* genes identified and cloned in all plant species belong to the NBS gene family, which is the biggest family of plant *R* genes. The NBS gene family encodes proteins with nucleotide binding sites (NBSs) and C-terminal leucine-rich repeats (LRRs) [25]. NBSs are capable of phosphorylating ATP/GTP to transmit downstream disease-resistance signals that are critical in fighting most pathogens. It is vital in order to constantly discover new *R* genes from the genetic materials that are still not completely applied to maintain an evolutionary advantage in the race between the disease and the host.

## 3. Effector

Effectors are broadly referred to as small-size proteins secreted by a pathogen which has the ability to interfere with the structure and effect of plant cells, encourage the pathogen’s infection, or elicit an immune reaction in the host, thus facilitating infection (such as virulence factors or toxins) and/or stimulating defense responses (such as avirulence factors or elicitors) [66,67]. Effectors often interfere with the initiation of defense responses by targeting host receptors or defensive signal components [68], such as inducing defense-related gene expression, activating downstream defensive signals, modifying specific proteins, and manipulating the signal of salicylic acid (SA), jasmonic acid (JA) and ethylene (Et) [69,70,71]. Effectors of fungal are characterized by low molecular weight (amino acid number < 300 aa), abundant signal cysteines at the terminus and poor sequence identity [72]. Low molecular weight and the N-terminal signal facilitate the secretion of effectors abundant in cysteine, fostering the persistent presence of effectors in the host and maintaining protein structure through disulfide bond formation [73]. Most effectors may be expressed and induce programmed cell death (PCD) in plants, or inhibit BAX/INF1/NEP1/NLP-induced PCD, thus interfering with plant immune responses and promoting pathogenic expression and expansion in host cells. During infection of rice tissues, *M. oryzae* secretes large numbers of effectors that stimulate the fungus to penetrate the epidermal cell, avoid identification by the host and reorganize host defense genes to promote pathogen growth and reproduction [74].

Effectors can be categorized in numerous patterns. On the basis of their molecular nature, effectors can be proteins [75], secondary metabolites [76], or small RNAs [77]. Based on the degree of damage they cause to the host, effectors are separated into Avr effectors and Non-Avr effectors. Avr effectors, an individual category of proteins generated via the avirulence (Avr) genes, can potentially be identified through associated R proteins, resulting in the identification of a particular race [78]. Effectors also can be divided into two different categories: apoplastic effectors and cytoplasmic effectors, dependent on where in the host plant they react. Apoplastic effectors are secreted outside the plant cell membrane and bind and interact with extra-plasma target proteins or cell membrane surface receptors, whereas cytoplasmic effectors are released into the cell and bind to corresponding intracellular proteins [79]. Some of these effectors, often referred to as “core genes”, can be found throughout the genomes of several closely or erratically related species [80]; others can only be discovered in particular strains or isolates of a certain species [81]. Based on the type of interaction or host specificity, effectors can be categorized accordingly. For instance, some effectors contribute to both plant–microbe and microbe–microbe interactions, while others are limited to a single interaction form [82].

### 3.1. AVR Effector

An increasing number of effectors are being identified in the rice and *M. oryzae* interaction network. In *M. oryzae,* there are 40 *AVR* genes which have been reported to be genetically analyzed [83], including 14 *AVR* genes (*AVR-PI54* [41], *AVR-PI9* [29], *AVR-PIA* [17], *AVR-PIB* [26], *AVR-PII* [17], *AVR-PIK/KM/KP* [17], *AVR-PIZT* [31], *ACE1* [84], *AVR-PITA* [51], *AVR1-CO39* [85], *PWL1* [86] and *PWL2* [87], while *MoHTR1* and *MoHTR2* [19] have been cloned (Table 2) [24]. The conventional R-Avr pairing proteins that have been proven to interact directly with one another include Pi-ta/Avr-Pita, Pik/Avr-Pik, Pia/Avr-Pia, Pi-CO39/Avr-CO39, and Pi54/Avr-Pi54. However, Piz-t/Avr-Piz-t and Pii/Avr-Pii maintain an indirect relationship with one another [71]. In addition to the one-on-one interaction mode, there are also two other types of interactions. One type is where different R proteins target the same Avr protein, just like Pik-1 and Pik-2 interact with Avr-Pik [88], and this phenomenon can also be found in *MoHTR1* and *MoHTR2*, which have been shown to associate with the same target protein, exhibiting a similar mechanism [19]. In another type of interaction, a single R protein complex is able to recognize two different Avr proteins. For example, *RGA4* and *RGA5* heterodimers function as NLR proteins, which interact with Avr-Pia and Avr-CO39, respectively [47,89].

#### 3.1.1. Avr-Pi54

“Tetep” Indica rice was employed for cloning the dominant *R* gene *PI54*, which was later confirmed to offer broad-spectrum resistance to *M. oryzae* [91]. Zinc finger and LRR domains, which are essential for ligand recognition and binding, have been discovered in an *R* gene *PI54* [92]. During the secretion of pathogen, Avr-Pi54 is predicated to incorporate cystine disulfide linkages to offer it stability [41]. An instantaneous relationship between Pi54 and Avr-Pi54 proteins was revealed through in silico docking and yeast two-hybrid assay. A transient expression experiment in *Nicotiana benthamiana* offered evidence that both *PI54* and *AVR-PI54* were able to cause hypersensitive cell death in a heterologous system when presented at the same time [41,93]. New research proposed that, in the absence of *PI54*, the Avr-Pi54 can translocate to the host cell’s nucleus, where it may act as a modulator of gene expression [94]. However, the transportation of Avr-Pi54 to the nucleus is impeded when the Pi54 protein is present in the host cell, due to Pi54 directly interacting with the Avr-Pi54, preventing any further movement and simultaneously inducing an HR-mediated resistance reaction [94]. The Pi54 alleles modify the physicochemical characteristics, structure of the LRR domain and protein, and the global free minimum energy of both resistant and susceptible alleles through nucleotide polymorphism. These differences also affect the relationships within Pi54 and Avr-Pi54 [93].

#### 3.1.2. Avr-Pi9

Avr-Pi9, an effector corresponding to the *R* gene *PI9* that encodes the NBS-LRR (nucleotide-binding site plus leucine-rich repeat) protein, was discovered using comparative genomics [29]. Avr-Pi9, which encodes a small secreted protein that is prominently expressed at the beginning of rice blast, seemed to be localized in the BIC (biotrophic interfacial complex)and distributed into the host in the process of invasion [29]. In *M. oryzae*, the absence of *AVR-PI9* failed to result in any observable abnormalities relating to either development or pathogenesis [29]. The most recent study discovered an entirely novel strategy, by which Avr-Pi9 undermines the immune reaction of the host and accelerates infection by targeting the OsRglg5, which belongs to a RING E3 ubiquitin ligase of rice [95]. Moreover, Avr-Pi9 directly targets Anip1 (Avr-Pi9 interacting protein 1), which likewise binds to Pi9 and functions as a negative regulator of blast resistance. OsWrky62, interacting with Anip1 in rice, is a beneficial regulatory factor for blast fungal resistance in a rice background without Pi9, and Avr-Pi9 may contribute to OsWrky62 degradation by preserving the stability of Anip1, reducing the host immune response. However, when Pi9 has been activated, the entire system undergoes significant modifications so that Pi9 can also stabilize Anip1, but at this point both Anip1 and OsWrky62 are able to combine with Pi9 [96]. Additionally, Pici1, a deubiquitinase that stabilizes methionine synthetases to enhance methionine-mediated immunity, is targeted for degradation by Avr-Pi9 to suppress PTI and further disrupt host immunity [97].

#### 3.1.3. Avr-Pia

Pia, initially discovered in the Japanese rice cultivar *Aichi-asahi*, was targeted by Avr-Pia to induced HR in the infection of *M. oryzae* [47]. The *AVR-PIA* encodes a minor secretory protein (85 aa), with 19 amino acids at the N-terminus, proposed to form the secretion signal that lacks any recognized protein domains [17,98]. Avr-Pia and Pia activation can cause programmed apoptosis in rice; the mutation in *AVR-PIA* is able to cause it not to be recognized by the corresponding disease resistance gene *PIA*, leading to pathogenicity [17]. In order to regulate Pia resistance and perceive Avr-Pia, *RGA4* and *RGA5* are two resistance genes positioned close to each other at the location of Pia, both of which are essential to mediate resistance to *M. oryzae* [47]. In both rice protoplasts and *N. benthamiana*, the presence of *RGA5* inhibits the Avr-independent cell death that *RGA4* causes. Once detecting the pathogen effector Avr-Pia’s direct binding to *RGA5*, repression is lifted and cell death ensues [89]. Avr-Pia is a cytoplasmic effector that is expressed at the beginning of appressorial differentiation and is transported to the BIC before entering the host’s cytoplasm, as evidenced by the co-localization with Pwl2, which is the typical representative of a cytoplasmic effector [99].

#### 3.1.4. Avr-Pib

The gene *AVR-PIB*, which provides resistance to hosts expressing the *R* gene *PIB*, has been isolated through map-based cloning. There is a 75-residue protein featuring a signal peptide in *AVR-PIB* [26]. Through the application of molecular analysis, four distinct *AVR-PIB* haplotypes were detected in the isolates from *MBYZ* (a susceptible elite cultivar). Different from *AVR-PIB-AP1-1* and *AVR-PIB-AP1-2*, *AVR-PIB-AP2* and *AVR-PIB-AP3* are virulent for *PIB* [100]. The investigation of sequence variations in the *AVR-Pib* gene in field isolates from the *Philippines* revealed that the Pot 3 transposon in *Avr-Pib* generated isolates with Pib-virulence. As a result, three distinct Avr-Pib haplotypes were produced, due to the three distinct Pot 3 insertions found in the genome [101]. Avr-Pib’s crystal structure suggested that it shares a lot of structural similarities with other *M*. *oryzae* Avr and ToxB (MAX) effectors. By comparing the structures of different MAX effectors, an individual positively charged patch (PCP) consisting of three portions of residues connected by a β-sheet was found on the surface of Avr-Pib [102]. These PCP residues have been determined to be fundamental to the function of avirluence and the nuclear localization of Avr-Pib in the host cells by site-directed mutagenesis and functional investigation [102]. Attention to this prevents the development of Pib homodimers and maintains Pib’s ineffective state; SH_3_P_2_ (a protein with an SH_3_ domain) binds to the CC domain of Pib to create heterodimers within a normal situation [103]. Nevertheless, once *M. oryzae* establishes an invasion into the host, SH_3_P_2_ preferentially chooses to interact with Avr-Pib instead of Pib, leading to the conversion of SH_3_P_2_-Pib heterodimers to Pib homodimers, and eventually causes ETI [103]. The avirulence of Avr-Pib depends on its nucleus localization [102]. Two WRKY transcription factors, *OsWRKY62* and *OsWRKY76*, and the effector Avr-Pib translocate to the nucleus by the interaction with OsImα1s (a rice importin) through novel nuclear localization signals to negatively regulate defense responses [104].

#### 3.1.5. Avr-Pii

*PII*, a rice resistance gene, encodes a pair of NLR proteins of the CC-NB-LRR type [105]. In *M. oryzae*, the small secreted protein (70 aa) which corresponds to Pii, is termed Avr-Pii, which has no known protein homologs [17]. In rice cells, Avr-Pii binds to OsExo70-F2/F3, a pair of rice Exo70 proteins thought to be involved in exocytosis, to generate a 150 kDa complex. Pii performance was shown to be impaired when OsExo70-F3 levels were decreased but not OsExo70-F2, indicating that OsExo70-F3 has a unique role in Pii-dependent resistance. The relationship that exists between Avr-Pii and OsExo70-F3 appears to be essential in the resistance induced by Pii, demonstrating OsExo70′s potential function as a decoy or assistant in Pii/AVR-Pii interactions [106]. Innate defenses against the rice blast fungus were hampered by the absence of *Os-NADP-ME2*, which performs as a resistant gene in rice cultivar. When a susceptible rice cultivar is infected, *M. oryzae* secretes Avr-Pii through the BIC, where it reacts with *Os-NADP-ME* to prevent ROS burst by inhibiting NADPH (an enzyme associated with ROS production) synthesis [107].

#### 3.1.6. Avr-Pik/km/kp

A couple of NLR receptors in rice, Pik-1 and Pik-2, serve as important elements to recognize the *M. oryzae* effector Avr-Pik, which is a secreted protein with a signal peptide at its N-terminus [38,108,109]. Pik-1 comprises an unconventional integrated HMA (heavy-metal-associated) domain which binds Avr-Pik straight away, to induce plant immunity [109]. There are five known and cloned Pik-1 alleles, with the bulk of them found in and around the integrated HMA domain, and the host targets of Avr-Pik are also HMA-domain-containing proteins [36,56,108,110]. The isolates of *M. oryzae* contain six alleles of *AVR-PIK* (*AVR-PIK*-*A*/-*B*/-*C*/-*D*/-*E*/-*F*), which diversify by only five amino acid positions [17,108,111,112]. Each of above polymorphic amino acids are all found near the Pik-1-HMA interface, which illustrates that they are all adaptable [109]. Among the other alleles, the most prevalent one is *AVR-PIK-D* [88]. In a separate investigation, a yeast two-hybrid assay identified four N-terminal HMA domain-containing proteins (OsHipp19, OsHipp20, OsHpp03, and OsHpp04) as Avr-Pik-D interactors [113]. Among them, *OsHIPP19* and *OsHIPP20* belong to the HIPP family, which contains heavy-metal-associated isoprenylated plant proteins [114,115]; however, *OsHPP03* and *OsHPP04* are associated with the HPP (heavy-metal-associated plant protein) family [113]. Interesting, Avr-Pik-D engages with the HMA domain presenting in OsHipp19, with a high affinity, and their interactions are more intimate than those between Avr-Pik-D and the HMA domain of Pik-1 alleles [113]. Meanwhile, the close association between OsHipp19 and Avr-Pik-D is not exclusive, and can also be observed in other Avr-Pik variants [113].

#### 3.1.7. Avr-Pita

Pita/Avr-Pita is the first pair of rice-resistant proteins to be associated with the non-toxic rice blast fungal protein directly, according to reports [51]. The original name of Avr-Pita has been modified to Avr-Pita1, due to research by Khang et al., which found many *AVR-PITA* gene homologs are present in genomes of multiple distinct isolates, and constitute a multigene family [116]. The avirulence gene’s activation is preserved by the putative neutral zinc metalloprotease, Avr-Pita1, which is composed of 223 aa and is located near a telomere [51,116]. Avr-Pita1 has an N-terminal signal peptide that is thought to be recognized by the NBS-LRR of the Pita, and is important for stimulating the immune response of the rice cell [51,117]. During infection, Avr-Pita1 builds up in the BIC structure before being transferred into host cells by IH [2]. Random mutations, insertions or deletions in *AVR-PITA1* can still successfully infect the host and produce pathogenicity [51]. The cytochrome c oxidase (COX) assembly protein OsCox11, an essential administrator of ROS in rice mitochondria, interacts with Avr-Pita1 to target the host mitochondria [118], and the activity of COX was boosted by overexpressing *AVR-PITA1* or *OsCOX11* [118].

#### 3.1.8. Avr-Pizt

The *AVR-PIZT* gene of *M. oryzae* triggers immunity in rice through the association with *PIZT* resistance gene, following a gene-for-gene pattern. Avr-Pizt is a cytoplasmic effector which prevents *N. benthamiana* cells from dying through anaphylactic reactions caused by BAX, suggesting that it may be involved in eliminating the plant defense response [119]. Avr-Pizt, which is released to BIC and shifted into rice cells, encodes a putative polypeptide (about 108 aa) with a secretion signal at its N-terminus [120]. There is an interaction between Avr-Pizt and *APIP6*, *APIP10* (two of the RING-type ubiquitin E3 ligases), contributing to Avr-Pizt regulating the host defense response by suppressing their activity and stimulating their degradation [120,121]. Intensive study found that inhibition of *APIP10* by Avr-Pizt resulted in an increased accumulation of *OsVOZ1* and *OsVOZ2*, which promoted Pizt transcription and translation, stimulating a strong Pizt-dependent ETI, restricting the invasion between *M. oryzae* containing *AVR-PIZT* and rice involving *PIZT* [122]. Moreover, Avr-Pizt interacts with the bZIP transcription factor *APIP5* in the cytoplasm and inhibits its transcriptional activity to facilitate blast fungus entry into the dead-body nutrition stage [123]. Avr-Pizt also competes with OsCipk23 (a cytosolic protein kinase) to cooperate with the potassium channel OsAkt1 to regulate the expression level of the K channel [124]. Avr-Pizt additionally collaborates with *APIP12*, which functions as the nucleoporin protein, to weaken the defensive responses of rice [125]. Recent studies verify that the lysine residues in Avr-Pizt are crucial for initiating the immunity mediated by Pizt against *M. oryzae*, and they also manage the generation of ROS and defensive reactions regulated by OsRac1 [126]. Recent research suggested that Avr-Pizt structurally mimics *ROD1* (resistance of rice to diseases 1), facilitating H_2_O_2_ diminution via triggering activation of catalase CatB, and that Rod1 and Avr-Pizt belong to a common cascade of protein degradation and ROS destruction. This discovery confirms the theory that effector of fungi has abused the immune reaction provided by the host protein [127].

#### 3.1.9. Ace1

Genetic and molecular research on rice varieties containing the *R* gene *PI33* has firmly established a connection between *ACE1* (avirulence-conferring enzyme) and the avirulent phenotype of strain Guy11 [90,128]. Rice varieties with the *R* gene *PI33* are resistant to *M.oryzae* strains or transformants that express functional *ACE1*, but isolates or mutants lacking *ACE1* can infect like resistant species, indicating that the recognition of Ace1 in rice blast fungus by Pi33 can prevent *M.oryzae* from infecting rice [90]. Interestingly, the avirulence of Ace1 is eliminated and avoids recognition by resistant plants when a critical amino acid in the catalytic site of the β-ketoacyl synthase domain is altered, revealing that its biosynthetic activity is required [90]. Additionally, *ACE1* is one of the essential members of a group of genes participating in secondary metabolism that are unique to infections [129], exclusively expressed only during penetration instead of at any additional period in *M. oryzae* [130]. Since Ace1 is not released into the host cells and is just located in the cytoplasm of the appressoria, it could potentially not be an effector [130]. But it is interesting that the avirulence signal detected through Pi33 fails to involve the Ace1 protein; instead, it involves a secondary metabolite that Ace1 synthesizes [90]. The hybrid PKS/NRPS (polyketide synthase/nonribosomal peptide synthase), produced by *ACE1*, encodes various enzymes associated with the synthesis of secondary metabolites that lack secretory properties. This phenomenon is particularly in opposition to most of the identified *AVR* genes [90]. Since *ACE1* in *M. oryzae* is under very strict temporal and cellular type-specific regulation, the avirulence molecule has not yet been isolated or purified. The development of novel compounds with great potential for use in crop protection will be aided by understanding of the *ACE1* compound. Researchers have increasingly concentrated on the homologous expression of different fungal *PKS/NRPS* genes in *Aspergillus oryzae* from gene clusters with established functions, and have utilized this technique to identify unidentified chemical products of cryptic pathways [131,132,133].

#### 3.1.10. Avr-CO39

*AVR-CO39*, an avirulent gene, was first discovered in *M. oryzae* strains 4091-5-8 and 2539 [134]. The 1.06 kb fragment on chromosome 1 of *M. oryzae* was found to be associated with the *AVR1-CO39* locus [85,135]; *AVR1-CO39* encodes a little secretory protein of 89 aa and no homologs that is only produced at an early and biotrophic stage of infection [49]. *AVR1-CO39* is secreted by rice protoplasts and re-enters the cytoplasm through unidentified mechanisms, indicating that its translocation into rice cells is independent of fungal factors [49]. *RGA4* and *RGA5*, two rice genes that encode NB-LRR proteins, are essential for acknowledgment of the *M. oryzae* effector Avr-CO39. In contrast to *RGA5*, which serves as an effector-binding receptor and regulator of *RGA4* signaling activity, *RGA4* works as a constant trigger of apoptosis [136]. To repress Rg4-mediated cell death without the presence of *AVR1-CO39*, Rga5 produces a heterocomplex with Rga4 [137]. Recently, the rice immune receptor *RGA5A_S* has been crystallized with effector *AVR1-CO39* using a combination of mixture and tandem strategies, which is contributing to our understanding of how *R* proteins recognize effectors [137].

#### 3.1.11. Pwl

The first Pwl effectors to be identified, Pwl1–Pwl4, are members of a small, rapidly developing family of effectors rich in glycine that confers avirulence on finger millet and weeping lovegrass but shows zero impact on rice. *PWL2* is a typical representative of the *PWL* gene cluster (*PWL1–4*) [87]. Pwl1 and Pwl2 normally contribute to the pathogenesis of rice blast, whereas Pwl3 and Pwl4 play no roles in this process [86]. Applying a map-based cloning strategy, Pwl2 is the first host-associated, non-toxic gene isolated from the Guy11 of *M. oryzae*. It expresses a 16 kDa glycine-rich, hydrophobic secretion protein [87]. Besides being released to BIC during plasmolysis, Pwl2 has also been discovered nearby without penetration into rice cells, concentrating the cytoplasmic signal [138]. Pwl2 expression was enhanced in the appressorium following invasion of a living rice cell, but it dramatically decreased in the highly branched hyphae when the first penetrated rice cell collapsed. This result suggested that the expression of Pwl2 is related to the sequential biotrophic invasion of the rice cell [139]. Since once the hyphae expand into living neighboring cells expression of *PWL2* boosts once more, therefore, for the fungus to express *PWL2*, it must enter the living cells of either the host rice or the non-host onion [139].

#### 3.1.12. MoHtr1 and MoHtr2

*MoHTR1* and *MoHTR2*, which stand for *M. oryzae*’s host transcription reprogramming 1 and 2, are cytoplasmic effectors that produce polypeptides with 198 and 110 amino acids, respectively [19]. *MoHTR1* and *MoHTR2* are implicated in plant immunity by localizing to the host nucleus [19]. These nuclear effectors feature a C_2_H_2_ zinc finger domain that combines with the promoter region of their object genes during *M. oryzae* invasion to suppress the activity of those genes. Interestingly, the *HTR* inhibition effect on the immunity-associated gene can resulted in two outcomes: firstly, increased resistance to the disease *Cochliobolus miyabeans*, and secondly, enhanced sensitivity to semi biological diseases such as *M. oryzae* and *Xanthomonas oryzae* pv *oryzae* (*Xoo*) [19].

### 3.2. None-AVR Effector

Avirulence factors, elicitors, PAMPs, toxins, and degradative enzymes are all accounted for in the broader category of effectors [66]. In addition to 14 Avr effectors, more than 37 None-Avr effectors have been functionally characterized, including 4 secreted proteins linked with biotrophy, 8 secreted proteins essential to pathogenicity, 13 proteins which reduce plant apoptosis, and 12 proteins that trigger apoptosis (Table 3).

#### 3.2.1. Bas Proteins

Four fungal biotrophy-associated secreted (*BAS*) proteins were confirmed a decade ago [140]. Bas effectors exhibit an intriguing variety of localizations in the cytoplasm and apoplast [140]. Similar to other recognized avirulence effectors, the Bas1, along with Bas2, preferentially gather in the BIC. Bas3 also exhibits another localization which is adjacent to cell wall connection points, and Bas4 is a putative EIHM (extra-invasive hyphal membrane) matrix protein which is released and expressed on the external surface of the hyphae [140]. During the biotrophic phase, *BAS1*–*BAS4* are significantly expressed, while *BAS4* shows expression at a 61-fold greater level in the IH [140]. Eight of the one hundred and two amino acids that the *BAS4* gene codes for are cysteine residues [140]. A study assessed the contribution of Bas4 to *M. oryza*’s movement from the biotrophic to the necrotrophic phase. When the same blast strain is inoculated into susceptible rice cultivars that were pre-treated with the prokaryotic expression product of *BAS4*, the result displays more severe blast disease symptoms, increased biomass (such as conidian and fungal relative growth), and reduced expression levels of genes involved in rice pathogenicity than in PBS-pretreated leaves [158]. This indicates that *BAS4* is involved in the transition of rice blast fungus from the biotrophic to necrotrophic phase and alters rice defenses in vitro, making it easier for *M. oryzae* to infect rice [158]. Recent findings implied that variations in the location of *OsRBOHB* (oryza sativa respiratory burst oxidase homolog B) at the region of invasion enable the accumulation of substantial ROS concentrations near the IH of *M. oryzae*, inhibiting the activation and secretion of Bas4 and fostering immunity in rice [159].

#### 3.2.2. Mpg1 and Mhp1

It has been demonstrated that proteins from the fungal hydrophobin and cutinase categories are crucial for *M. oryzae*’s invasion of rice [4,160]. Small surfactant proteins known as hydrophobins can decrease the surface tension, which makes it easier for aerial hyphae and conidian to develop. Additionally, they wrap the spore’s membrane to lessen moisture retention and promote interactions with the surface [143]. The class I fungal hydrophobin, *MPG1*, encodes a small secreted, hydrophobic protein, which is abundantly produced when a plant is first infected, along with when symptoms of the disease begin to appear [141,161]. During the conidial generation and host invasion, the *MPG1* gene is managed by at least three channels of signaling (PMK1 MAPK, cAMP response, and nitrogen repression pathways), whose functions are achieved through involvement in three prominent cis-acting regions upstream of the *MPG1* gene [162].

*MHP1* is a hydrophobin that is a member of the class II group, according to the measurement of the hydrophobicity of amino acids. A typical 102-amino-acid fungal hydrophobin, with eight cysteine residues organized in the traditional manner, it is encoded by the *MHP1*, and approximately twenty percent of the amino acid sequence in *MHP1* resembles *MPG1* [143]. During colonization and conidiation, *MHP1* activity is strongly triggered, whereas it is scarcely perceptible during the development of mycelia [143]. Mutant Δ*mhp1* also show deleterious effects on fungal morphogenesis, including conidia formation, conidia germination, appressorium development, and infection of the host cell. Additionally, the organelles inside the cells of the Δ*mhp1* conidia are flawed, causing a rapid loss in their viability [143]. Assembling functional amyloid structures termed rodlets, *MPG1* connects laterally to create amphipathic, fibrillar layers, but *MHPI* develops amphipathic layers without fibrillar structure. When combined with *MPG1*, *MHPI*, which has a high surface activity, can prevent *MPG1* rodlet assembly. The pathogenicity of fungal cutinases, which can break down cutin, is crucial [163]. A layer of *MPG1* rodlets is capable of boosting cutinase activity once it has been produced by tracking the protein to a surface or by enhancing the activity of the enzyme through conformational changes [164].

#### 3.2.3. Emp1

The open reading frame of *EMP1* (extracellular matrix protein 1) consists of six hundred and eighty-five nucleotides, which code for twenty-seven amino acids. The protein encoded by *EMP1* has an approximate molecular weight of 20.5 kDa, carries an N-terminal secretory signaling sequence of eighteen amino acids, an has four potential N-glycosylation sites [142]. Northern blot indicates that *EMP1* expressions increased at the time of appressorium formation rather than during vegetative development. In the mutant Δ*emp1*, decreased appressorium generation and pathogenicity have been observed, but neither mycelial development rate nor conidiation capability were affected. The results presented imply that *EMP1* is essential for the development of appressoria and for the pathogenicity of *M. oryzae* [142].

#### 3.2.4. Slp1

The effector Secreted LysM (lysin motif) Protein1 (Slp1) contains two LysM domains, which are microscopically localized between the infested mycelium and the EIHM, and belongs to the apoplastic effector [144]. Slp1, which performs plays an indispensable part in the pathogenesis of rice blast, decreased the plant’s defensive reaction to chitin involving the accumulation of ROS and the expression of plant defensive proteins through interacting with chitin by competing with rice CEBiPs [144]. Silencing the *CEBiP* in Δ*slp1* reinstated the pathogenicity of Δ*slp1*, indicating that the Slp1 works as an critical factor in the maintenance of the plant’s defensive reaction to *M. oryzae* [144]. The insect pathogen *Beauveria bassiana* contains extracellular LysM proteins that are virulence components. Intriguingly, complementation with the Slp1 from *M. oryzae* was able to totally repair the virulence deficiencies of two *B. bassiana* mutants, Δ*blys2* and Δ*blys5* [165]. *ALG3* encodes an enzyme that participates in the N-glycosylation of proteins, an a-13-mannosyltransferase, and the deficiency of *ALG3* led to diminished virulence and blocked the development of secondary IH. There are three N-glycosylation sites in Slp1, and every one of them is controlled by Alg3. N-glycosylation, essential for the function of effector proteins, is necessary for preserving the stability of the protein and chitin-binding activity of Slp1 [166].

#### 3.2.5. Mc69

*MC69*, a protein containing 54 amino acids and presented in BIC, is projected to contain a potential N-terminal secretion signal peptide and to be crucial for *M. oryzae* pathogenicity, IH formation, and appressorial penetration. In Mc69, there are 38 amino acids which are predicted to show functional areas that are unknown [145]. The expression of *MC69* has been detected in mycelia, conidia, and each phase of the infection. *MC69* mutants were unable to enter plant cells and do not effectively interact with the host [145]. The pathogenic activity mediated by *MC69* has been demonstrated to depend on the pair of cysteine residues (C 36 and C46) which are conserved amongst the Mc69 homologs and may be responsible for the production of disulfide bridges [145].

#### 3.2.6. Chia1

MoChia1 (chitinase 1), a recently identified effector, has been demonstrated to interact with chitin and hinder chitin-triggered immunity via a mechanism which is like Slp1 [18]. *MoCHIA1* contains 397 amino acids, among which there is an N-terminal signal peptide. As an invaluable chitinase that is necessary for *M. oryzae*’s growth and development, if *MoCHIA1* is silenced it will drastically decrease the fungus’ pathogenicity [18]. With an application of an inducible promoter in rice, the expression of *MoCHIA1* increases the host’s resistance to *M. oryzae*, confirming that *MoChia1* has the capacity to instigate host defense reactions [18]. Overexpressing *OsTPR1*, a rice tetratricopeptide repeat protein, caused an increase in the content of ROS during the invasion of *M. oryzae*, and *OsTPR1* was able to bind to MoChia1 in the rice apoplast to promote the accumulation of free chitin and the restoration of the defensive reaction [18].

#### 3.2.7. MoAo1

By managing the apoplast redox state, apoplastic ascorbate oxidases (AOs) have been shown to be crucial in generating ROS, to impact natural host immunity. There is an N-terminal secretion signal along with three AO domains contained in a 603-aa peptide which is encoded by *AO1*, implying that Ao1 has AO activity [167]. During the infection, MoAo1 is expressed and secreted to the EIHM, so MoAo1 belongs to the apoplastic effector [146]. It has been found that polymorphic mutations of *MoAO1* influence its enzymatic functions and the virulence of *M. oryzae* via the disruption of the redox situation in the apoplast of rice [146]. Rice avoids being recognized by *M. oryzae* through polymorphic variations in *OsAO3* and *OsAO4.* By limiting the enzyme production of rice *OsAO3* and *OsAO4*, MoAo1, which is constrained by MoSwa2, performs an essential function in the redox state equilibrium of the host apoplast in *M. oryzae* [146]. Each of the polymorphic types of *MoAO1*, OsAO3, and OsAO4 regulates the relationship between pathogenic virulence and rice immunity [146].

#### 3.2.8. Rrf1

For a focal BIC to develop in *M. oryzae*, a particular gene called *RBF1*, Required-for-Focal-BIC-Formation 1, is needed. Rbf1, like other *M. oryzae* cytoplasmic effectors, aggregated in the BIC and moved into the rice cytoplasm. The only time the fungus becomes infected on tissue from living plants is when the expression of *RBF1* in the appressoria and in the IH becomes activated. Fluorescence imaging verified that the production of *RBF1* was raised on every occasion of fungus penetrating the cell wall of the host [147]. While substantially lacking in pathogenicity to rice leaves, mutant Δ*rbf1* is nevertheless able to keep propagating in rice plants treated with abscisic acid or lacking in salicylic acid. Inoculating Δ*rbf1* causes necrosis and elevates the activity of defense-related genes in rice leaves, which leads to an increased amount of diterpenoid phytoalexin accumulation compared to wild type [147]. The mutant Δ*rbf1* has resulted in a reduced transfer efficiency of cytoplasmic effectors, aberrant cytoplasmic effects distribution, and faulty IH differentiation [147].

#### 3.2.9. Iug4, Iug6 and Iug9

The pathogenicity of *M. oryzae* depends on the zinc finger protein called MoIug4 (isolate-unique genes), which acts as a transcriptional repressor to weaken host defenses by targeting the rice ethylene pathway [148,149]. MoIug4 is a 133 amino acid polypeptide with a C-terminal region that contains a zinc binding domain [148]. *OsEIN2*, produces a critical signal transducer in the Et pathway in rice, and MoIug4 combines with its promoter [148]. Iug4 has been demonstrated to have a greater affinity for the *OsEIN2* promoter region, and adversely affects the production of *OsEIN2*. Iug4, therefore, may have a negative impact on the plant’s immune reaction by disrupting the ET pathway [167].

The small secreted protein Iug6 with four Cys residues is similar to the known cysteine-rich proteins Bas2 and Avr-Pizt [149]. *IUG6* and *IUG9* have been identified in the BIC, and overexpressing them in rice inhibits the production of defense-related genes [149]. Functional characterization has shown that Iug6 and Iug9 have a critical influence on *M. oryzae* in the area of mycelium development, conidial production and virulence [149]. Additionally, overexpression of *IUG6* or *IUG9* in *M. oryzae* may reduce the level of expression of *PR1a* and *CHT1* in rice, which act as markers for SA and ET pathways, respectively [149].

#### 3.2.10. Nups

Nups are crucial parts needed for nuclear pore complex (NPC) assembly and are vital for plant immunity [125]. There are three non-specific proteins, Nup1/2/3 (MGG_07900/MGG_08024/MGG_04546), which have been discovered, and via a PVX-based high-throughput transient plant expression system it was found that every one of the Nup1/2/3 prevents BAX cell-death in *N. benthamiana* [149].

#### 3.2.11. MoHeg13

In *M. oryzae*, a variety of hypothetical effector genes (*MoHEG*s), categorized as early and late *MoHEGs* on the foundations of the maximal transcript abundance during colonization of barley, have been discovered using microarray research [150]. *MoHEG16*, from the early-expression group, has been demonstrated to be involved in colonization in the plant, whereas *MoHEG13*,compared with the late-expression group, has been determined to be a suppressor of NLP (necrosis- and ethylene-inducing protein 1 (Nep1)-like protein)-induced cell death [150]. Mutant analysis revealed that Late-*MoHEG* MoHrg13 is necessary for complete pathogenicity in *M. oryzae* and that the isolation strain which shows the absence of *MoHRG13* generates fewer symptoms than the wild type and has lower invasion at 24 hpi, the point at which the corresponding gene is expressed at the maximum [150].

#### 3.2.12. Spds

Using the techniques of transient expression, a serious of suppressors of plant cell death (*SPD*) effectors coming from *M. oryzae* were verified to be able to prevent apoptosis of the plant by Nep1 in *N. benthamiana*; there were ten of the eleven *SPD* genes which also inhibited cell death caused by BAX [151,168]. In the initial screening, only *SPD11* was confirmed to be incapable of preventing the cell death brought on by BAX [151]. Through the results of effectoromics screening, it was found that the most effective inhibition of apoptosis involving both Nep1- and BAX-mediated apoptosis was provided by *SPD1* and *SPD9* [151]. There are five of the eleven *SPD* genes which have already been determined to be either critical for *M. oryzae*’s virulence or which are believed to be inhibitors or the homologs to additional identified suppressors [151]. *SPD2*, *SPD4*, and *SPD7* show nucleotide polymorphism in the isolates. Among them, *SPD4* shows the maximum level of nucleotide diversity known in currently identified *M. oryzae*’s effectors, indicating that the gene might be under selection to avoid recognition by the host (Sharpee et al., 2017).

#### 3.2.13. MoHrip1 and MoHrip2

MoHrip1, an HR-inducing protein elicitor, is the first discovered in *M. oryzae* culture filtrate [152]. The first 16 amino acids of the small protein MoHrip1, which has 142 amino acids, function as a signal peptide [152,169]. During the penetration into *M. oryzae*, *MoHRIP1* activity is elevated, and it has the ability to initiate early phases of tobacco defensive reaction, such as the generation of hydrogen peroxide, the deposition of callose, and the alkalization within the extracellular media [170]. Real-time PCR results demonstrated MoHrip1 also stimulates the expression of numerous genes associated with signaling and disease. Additionally, the SA pathway seems to contribute to MoHrip1′s ability to enhance expression of defense-related genes [170]. In rice, transcriptional profiling confirmed that MoHrip1-treated seedlings have increased systemic immunity towards *M. oryzae* [170]. Moreover, preventive response of plant *M. oryzae* has been significantly improved via overexpressing the *MoHRIP1* gene in rice [171]. All these results collectively imply that MoHrip1 functions as a virulence factor that promotes microbial infection, as well as an elicitor of plant immunity [152]. A newly discovered elicitor protein (16.252-kDa) in relation to *M. oryzae*, MoHrip2 causes quicker tissue necrosis in leaf tissue from tobacco and additionally elevates rice seedling immunity to *M. oryzae* [172]. Another crucial early-signal molecule, NO, which is a special diffusible molecular mediator within animals, performs a crucial part in a variety of physiological events in plants as well, and it can also be activated by MoHrip2 when invading the tobacco cell [172,173]. As it is discovered in host apoplast during penetration and is released by the traditional ER–Golgi pathway, MoHrip2 functions as an apoplastic effector. MoHrip2 also decreases immune system activity, through the production of specific immunity genes and the generation of particular phytoalexins, working like a virulence element that promotes fungal invasion and proliferation [153].

#### 3.2.14. MoCdis

Applying data from the transcriptome on rice leaves that were inoculated with *M. oryzae*, 851 genes of *M. oryzae* encoding potential secreted proteins were discovered. Chen et al. transiently expressed 42 candidate secreted-protein genes in plants and discovered five cell death-inducing proteins (MoCdip1 to MoCdip5) in *M. oryzae* [174]; and each one of them can induced cell death in rice cells. The sequences of the five *MoCDIPs* differ significantly from one another. While *MoCDIP3* displays little resemblance to any known protein, *MoCDIP1/2/4/5* have closely associated homologs from *M. oryzae* or alternative microbes [174]. Five of the MoCdips are apoplastic effectors that are expressed heavily in the course of the infection. While *MoCDIP3*, *MoCDIP4*, and *MoCDIP5* activities were exclusively discovered in appressoria, the expression levels of *MoCDIP1* and *MoCDIP2* have been identified in both appressoria and mycelia, and they are expressed more strongly in mycelia [174]. Eight newly identified proteins, numbered from *MoCDIP6* to *MoCDIP13*, have recently demonstrated the capacity to cause apoptosis of the plant [154]. It is worth noting that *MoCDIP*s (at least *MoCDIP6/7/8/10/11* are dispensable for pathogenicity [154]. Along with the already identified *MoCDIP1*-*MoCDIP5*, the recently discovered *MoCDIP6*-*MoCDIP13* provide additional perspectives for deeper comprehension of the molecular process between host and pathogen.

Effector *MoCDIP4* is classified as part of the glycosyl hydrolase family 61, and encodes a polypeptide of 295 amino acids; it also has a fungal-like cellulose-binding domain [167]. For the purpose of alleviating rice immunity, *MoCDIP4* binds to the mitochondria-associated protein complex *OsDJA9*-*OsDRP1E* [174,175]. The DnaJ protein OsDjA9 shows an interaction with the dynamin-related protein *OsDRP1E* to enable its degradation, and *OsDRP1E* is involved in mitochondrial fission, in turn controlling the stimulation of subsequent immunological responses [175]. On the other hand, *MoCDIP4* fights with *OsDRP1E* for the opportunities to bind to *OsDjA9*, thereby resulting in an elevated content of *OsDRP1E*, a declined ROS production, and a suppression of an augmented immune response [175]. Overexpression of *OsDRP1E*, *MoCDIP4,* or *OsDjA9-*knockouting causes both shorter mitochondria and higher vulnerability to *M. oryzae* in transgenic rice [175]. Furthermore, increased mitochondria and more powerful resistance to *M. oryzae* result from the excessive expression of *OsDjA9* or the absence of *OsDRP1E* in transgenic rice [175]. The first effector to engage the host in the ER and modify the dynamics of the mitochondria is *MoCDIP4* in *M. oryzae* [167].

#### 3.2.15. Msp1

Cerato-platanin (CP) proteins are classified as secreted proteins, and there are four conserved cysteine residues which can be discovered in them [176]. CPs can act not only as virulence factors in fungi but also as defense elicitors in plants [176]. Members of the CP family, snodprot proteins have already been found in a broad spectrum of fungi [155]. Msp1, a homolog protein to snodprot1, is a secreted protein which has a molecular weight of about 12 kDa in *M. oryzae* [155]. It was discovered that *MSP1* performs an essential function in relation to the virulence in *M. oryzae*, which was identified by the decreased ability of the pathogen on barley leaves via inoculation with mutant Δ*msp1.* After being released into the rice’s apoplasts, the protein Msp1 is recognized by the outer membrane and causes autophagic PCD and PTI [155]. Additionally, Msp1 generates significant plant apoptosis in cells and contains the potential to activate plants’ defensive mechanisms when it is applied exogenously, revealing that preliminary treatment, along with Msp1, could serve as a PAMP contributor to boost host immunological reactions to infections caused by pathogens [177]. The phytohormones JA and ABA (abscisic acid), as well as one or more protein kinases, might regulate the *MSP1*-induced signal [177].

#### 3.2.16. Nlp

The Nep1-like proteins (NLPs) are a group of molecular patterns which are frequently found in different kinds of plant-associated microbial species. There are four *MoNLPs* genes: *MoNLP1*, *MoNLP2*, *MoNLP3*, and *MoNLP4* (the corresponding gene to MGG_08454, MGG_00401, MGG_02332 and MGG_10532, respectively), which have been discovered in *M. oryzae* [150,178]. Since the *MoNLP* family has been strongly conserved, it is likely that MoNlps are crucial to *M. oryzae*’s biological processes [156]. In *N. benthamiana*, the results of transient expression have shown that three MoNlps (*MoNLP1*, *MoNLP2*, and *MoNLP4*) caused apoptosis, along with the generation of ROS [156]. During the infection, all four MoNlp proteins were upregulated, with different induced features. There is a strong induction of *MoNLP2* at 8 hpi, which is rapidly downregulated thereafter; at various times during either initial or final infection, *MoNLP4* transcript levels are markedly increased; when chlorosis symptoms first appeared at 48 hpi, *MoNLP1* began to be elevated; in contrast, *MoNLP3* is upregulated at 96 hpi, a period which is relatively late in the infection, when the infected leaf tissue is collapsing and spores are visible [156]. Based on the various patterns of transcript accumulation, *MoNLPs* are probably implicated in both the biotrophic and necrotrophic processes of invasion. The *MoNLP* family, however, is not necessary for rice plant infection or growth under different stress situations [156].

#### 3.2.17. MoSm1

The gene of the CP family encodes the small-size (~150 aa) and cysteine-rich protein which is secreted by filamentous fungi, which have been indicated to be linked to the virulence of some plant pathogenic fungi [179]. Evidence suggested that CP from *Ceratocystis platan* is capable of triggering a number of structural and physiological defensive reactions, like plasmolysis, apoptosis, the formulation of phenolic compounds and phytoalexins, and the upregulation of an immunity gene in both host and nonhost species [180]. The CP protein from *M. oryzae*, which boosts rice resistance to infection, is encoded by the gene *MoSM1* [157]. *MoSM1* is constitutively expressed not just during the distinct phases of growth development, but also in the invasion of the host [180]. Transient expression of *MoSM1* in rice leaves causes a hypersensitive reaction and an elevated level of defense genes [157]. In tobacco leaves, *MoSM1* is transiently expressed and targets the plasma membrane. SA and JA accumulation levels were higher in *MoSM1-OE* (*MoSM1*-overexpressing) plants, and the expression of SA and JA signal-related controlling defensive genes was constitutively elevated [157].

## 4. Localization of Effector in Plant Cell

**Figure 3:**
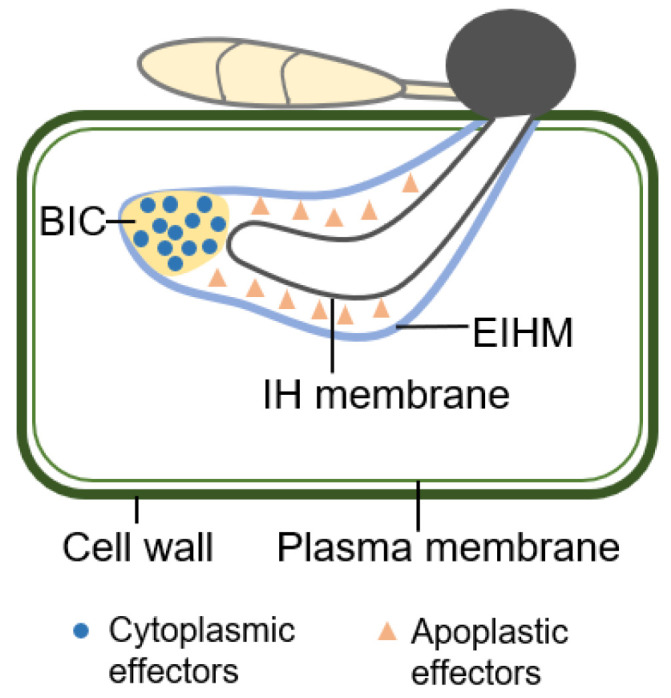
The construction of *M. oryzae* formed during infecting plant cell.

Effectors released by *M. oryzae* are capable of being classified into two distinct categories which are dependent on the location in which they are found within living cells: cytoplasmic effectors and apoplastic effectors. Cytoplasmic effectors including Pwl1, Pwl2, Bas2, Avr-Pizt, Iug4, Iug6, Iug9, MoHtr1 and MoHtr2 are gathered in the BIC (biotrophic interfacial complex), preferentially before being delivered into plant cells. A unique membrane-rich structure of plant origin, BIC is present in the location at the top of the pathogens’ primary IH (Figure 3) [2]. Following the inoculation at approximately 28–30 hpi, a single BIC develops in the first infected rice cell and subsequently expands to adjacent cells close to 44 hpi [181]. Each initial invading cell has only one BIC, whereas subsequent invading cells may have several BICs, one for each IH that penetrates the cell [182]. According to a hypothesis, BICs go through a three-stage phase of development, and function as the initial point of effector translocation, through the EIHM towards the host’s cytoplasm [2,10,183]. BICs complete three phases of formation and functional realization in the first cells to be invaded. In the first period, just one BIC develops at the tip of the filamentous primary IH; in the subsequent stage, the tip-BIC transforms into the “early-side BIC”, upon which the filamentous hypha differentiates into bulbous growth; during the final stage of development, the early-side BIC remains on the outside of the first bulb-shaped IH cell as a “late-side BIC”; at the same time, IHs keep expanding inside the rice cell [182]. Cytoplasmic effectors are secreted into the BIC by a unique mechanism combining the exocyst complex and t-SNAREs [10]. The blocking of the interaction between SNARE proteins prevents the normal formation of the BIC, inhibiting the secretion of the effector [184]. For example, the t-SNARE component MoSso1 interacts with MoSnc1, contributing to the establishment of BIC and the secretion of the cytoplasmic effector [184]. The *MoSSO1* deletion mutant in *M. oryzae* suffers impairments during BIC development, as well as pathogenic pathogenesis [10]. Additionally, the Qc SNARE protein MoSyn8 controls the secretion of cytoplasmic effectors but not apoplastic effectors [185]. 

Apoplastic effectors, such as Bas4, Slp1 MoAo1 and MoChia1, and others, are not connected to the BIC. They are distributed after secretion in the extracellular space between the EIHM (extra-invasive hyphal membrane) and the fungal cell wall. IH are encased in EIHM in *M. oryzae*, creating an interfacial space where apoplastic effectors can be regulated (Figure 3) [2,10]. Apoplastic effectors usually disperse, and are maintained in the EIHM compartment, which surrounds the entire IH and prevents them from casually entering host cells. Early IH growth is surrounded by the host plasma membrane as EIHM biogenesis begins. When IH differentiates into bulbous growth, however, the rebuilding of EIHM takes over [182]. The deficiency of EIHM integrity within *M. oryzae* penetration of rice is in sharp variance to the biotrophic pathogens, which shows that the integrity of the pathogen–plant membrane interface is preserved [2,11,186]. A recent investigation predicted that the disruption of EIHM occurs within the first-infected cell and before the rebuilding of biotrophy in the second-infected cell, undergoing three diverse infection stages (early-biotrophic, late-biotrophic, and transient-necrotrophic phases) [182]. De novo construction of EIHMs is likely to regulate host membrane dynamics, a function similar to other host–pathogen interactions [187,188]. However, the membrane source and trafficking mechanism for the assembly of the EIHM in the interaction between rice and *M. oryzae* are yet unknown. Rim15, a serine/threonine protein kinase of *M. oryzae*, was recently discovered to be necessary for biotrophic growth, maintaining biotrophic interfacial membrane integrity, and suppressing plant defense. It performs these functions by coordinating cycles of autophagy and glutaminolysis in invasive hyphae [181].

## 5. The Secretion System of Effector

During the infection of *M. oryzae*, at what point in time does the effector begin to secrete? It has been hypothesized that in *M. oryzae*, effector secretion begins at the bottom of the appressoria, prior to IH development [167]. Two diverse mechanisms for effector secretion have been recognized in *M. oryzae*, which is consistent with the localization of two distinctive kinds of effectors.

**Figure 4:**
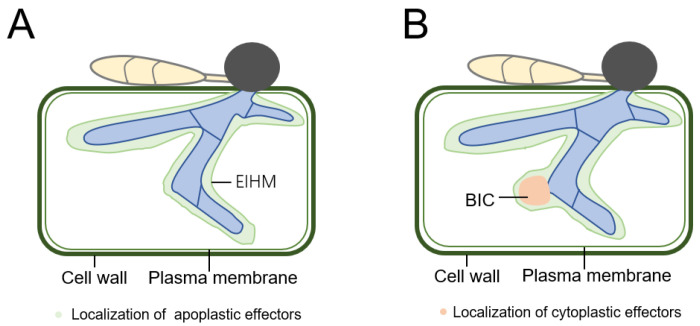
The located characteristics of effectors. (**A**) the located characteristics of apoplastic effectors; (**B**) the located characteristics of cytoplasic effectors.

In one secretion system, the conserved ER–Golgi secretory journey is employed to release apoplastic effectors into the extracellular space that exists between the cell wall of fungi and the extracellular hyphal membrane developed by plant cells (Figure 4A) [189]. Apoplastic effector secretion was blocked by a therapy of Brefeldin A, however, the cytoplasmic effector localization to the BIC was unaffected. Brefeldin A can block the operation of Golgi resulted in a restriction of the secretion of apoplastic effector [10,190]. Various post-translational modifications and ER–Golgi secretory pathway-related proteins are believed to be crucial for *M. oryzae* to secrete apoplastic effectors. For instance, the function of MoErv29 makes the virulence of *M. oryzae* more efficient, because it is the protein from the ER-derived vesicles which encourages the release of apoplastic effectors [191]. The proper distribution of the apoplastic effectors is impacted by MoSec61, a subunit of the translocation of the misfolded proteins out of ER [192]. The freshly synthesized proteins are exported from the ER via the COP II vesicles, as opposed to the COP I vesicles, which enable transport from the ER to Golgi [193]. MoSwa2, acts as a COP II uncoating factor and is essential for the secretion of effector and the development of IH [194]. Additionally, the vesicle transportation peptides actin and microtubules are merely essential for the release of apoplastic effectors and not for cytoplasmic effectors [195]. 

In the other system, the BIC, which is not reliant on the Golgi-dependent secretory pathway, is employed to deliver effectors into plant cells (Figure 4B) [195]. Instead of using the traditional ER–Golgi pathway, *M. oryzae* applies exoycst-mediated exocytosis to transmit and accumulate almost all cytoplasmic effectors in the BIC, before distributing them into the host cytoplasm [10]. In filamentous fungi, exocytosis performs essential functions in the establishment of cell polarity and the secretion of the effector, indicating that exocytosis plays an critical factor in morphogenesis and virulence [196]. Sec5 and Exo70 mediate the exocyst to control the secretion of cytosolic effectors, and a deficiency in these results in a markedly reduced virulence [10]. These results are evidence for the fact that the process of secretion of Pwl2, along with other cytoplasmic effectors, needs the exocyst to induce pathogenicity, and the mutant exocysts do not show the defect in the secretion of the apoplastic effector. Based on the above mechanism, secretion systems that provide proteins to various sites that are necessary for plant pathogenesis can be identified by exocyst-dependent and exocyst-independent pathways. The process that determines which secretion system will be employed by a specific effector is still unclear. Additionally, it is unknown whether exocysts support the secretion of the uncharacteristic effectors during disease in filamentous fungi. It is yet unidentified how apoplastic effectors relate to intracellular targets in plant cells or are identified by surface receptors. Furthermore, no particular protein pattern or sequence in the cytoplasm or apoplast has been discovered to localize them in plant cells after secretion.

## 6. Conclusions

One of the greatest threats to the globe’s rice harvest is rice blast. *M. oryzae* is also regarded as the most potent possible biological weapon, which attacks every part of the rice, from the roots to the panicles. On the other hand, although scientists and rice breeders have achieved some success in controlling rice blast fungus, they never dare to underestimate *M. oryzae* because of its amazing genomic plasticity, which enables it to modify itself by altering the host. Pathogens always have the potential to develop new virulent strains, thereby disrupting existing drug-resistance responses [197]. More research is required to better understand the molecular processes of pathogenicity and resistance, to develop strategies to combat the pathogen’s adaptive potential, and to examine the relationship between *M. oryzae* and rice. A crucial step in the penetration of *M. oryzae* is the selection of the effector to the target host. Corresponding to a variety of immunity systems and reactions, effectors perform a series of functions to respond, including controlling transcription or reducing/inducing defense. At least 851 genes for small secreted proteins with the potential to function as effectors have been identified in *M. oryzae*, although relatively few of them have been demonstrated to be pathogenic [198]. Therefore, further characterization of these effectors is of paramount importance for a better understanding of the regulatory mechanism resistant to the host defensive response in the period of *M. oryzae* infection. Effectors exhibit a significant degree of sequence variability, driven by the concurrent evolutionary competition between pathogens and the host. Currently, the identification of potential effector proteins is a formidable task, due to their distinctive sequence characteristics. The majority of fungal effectors are small secreted proteins lacking known conserved domains or motifs, and are presumably functionally redundant, due to the absence of visible virulence phenotypes in single deletion strains. Additionally, there is a scarcity of sequence similarities among fungal effectors. Consequently, it remains a challenge to predict their function within the host cell.

In recent years, significant progress has been made in cataloging novel secreted effectors in *M. oryzae*. For instance, computational structure prediction and machine learning approaches have enabled more precise descriptions of fungal effector functions. Nevertheless, novel high-throughput techniques for in-depth authentication and characterization of new candidate effectors are needed, to keep pace with the identification. In spite of the progress made so far, there are still numerous emergency problems with the effectors of *M. oryzae* that have yet to be answered. What job is the effector supposed to carry out once it enters the host cell? Which host channels are most frequently used by the effectors? Why do *M. oryzae* effectors have such an elevated degree of redundancy? Do they have comparable interactors in common, or are they possibly needed at various phases of infection to target the same host protein or a different one, to evade identification as the infection develops? An important line of investigation to consider is whether a particular effector has the same target in different hosts. The question of whether a specific effector shares the same target in multiple hosts is a crucial one to take into consideration. Therefore, additional research is needed to gain a more complete and sophisticated comprehension of the critical pathogen infection processes that control the outcome of plant–pathogen interactions. In future research, we can deepen our research on effector proteins by focusing on the following aspects: (1) studying the unique sequence and structure of the effector via the application of nuclear magnetic resonance and protein crystallization; (2) using genomics resources, bioinformatic techniques, and transcriptomic and proteomic resources to clarify the mechanisms behind the frequent emergence of new races of *M. oryzae*, creating a regulatory network between rice and *M. oryzae* and advancing our understanding of host–pathogen interactions; and (3) CRISPR-Cas tools for single-gene and multi-gene knockout, as well as precise editing through base editors and prime editors, enabling us to decipher a large number of host–pathogen interactions.

There are still many challenges for effector research in future; therefore, researchers need to continuously explore and innovate to overcome these challenges and promote the in-depth development of research on effector proteins and *M. oryzae*.

## Figures and Tables

**Table 1:** Cloned blast resistance (*R*) genes/alleles in rice.

*R* Gene	Encoding Protein	Donor	Reference	*R* Gene	Encoding Protein	Donor	Reference
*Pi-b*	NLR	Tohoku IL9	[26,27]	*PiPR1*	NLR	-	[28]
*Pi9*	NLR	75-1-127	[29]	*Pi2*	NLR	Jefferson	[30]
*Piz-t*	NLR	Zenith	[31]	*Pi50*	NLR	Er-Ba-zhan (EBZ)	[32]
*Pii*	NLR	Hitomebore	[17]	*Pizh*	NLR		[33]
*Pik*	NLR	Kusabue	[17]	*Pigm*	NLR	Gumei4	[34,35]
*Pik-p*	NLR	K60	[36]	*Pi-d2*	B-lectin receptor kinase	Digu	[37]
*Pikm*	NLR	Tsuyuake	[38]	*Pi-d3*	NLR	Digu	[39,40]
*Pi54*	NLR	Tetep	[41]	*Pi25*	NLR	Gumei2	[42]
*Pi54rh*	NLR	*Oryza rhizomatis* (nrcpb 002)	[43]	*Pid3-A4*	NLR	A4 (*Oryza rufipogon*)	[44]
*Pi54of*	NLR	*Oryza officinalis* (nrcpb004)	[45]	*Pi36*	NLR	Kasalath	[46]
*Pia*	NLR	Sasanishiki	[17,47]	*Pi5*	NLR	RIL260	[48]
*Pi-CO39*	NLR	CO39	[49]	*Pi56*	NLR	Sanhuang-zhan No. 2	[50]
*Pi-ta*	NLR	Yashiro-mochi	[51]	*Pb1*	NLR	Modan	[52]
*Pish*	NLR	Nipponbare	[53]	*Pike*	NLR	Xiangzao 143	[54]
*Pi35*	NLR	Hokkai 188	[55]	*Pik-h*	NLR	K3	[56]
*Pi37*	NLR	St. No. 1	[57]	*Pi1*	NLR	C101LAC	[58]
*Pi64*	NLR	Yangmaogu	[59]	*Pi65*	LRR- RLK	GangYu 129	[60]
*Pit*	NLR	K59	[61,62]	*Ptr*	ARM repeat domain protein	M2354	[63]
*Pi21*	Proline-rich metal binding protein	Owariha-tamochi	[64]	*Pi63*	NLR	Kahei	[65]

NLR: nucleotide-binding leucine-rich repeat; Chr. No: Chromosome number.

**Table 2:** List of *M. oryae* Avr cloned effectors.

*AVR* Gene	Protein Size	Chr. No	Effector Type	Cognate *R* Gene	Site of Secretion	Reference
*AVR-PI54*	153	4	ToxB-like	*Pi54*, *Pi54rh*, *Pi54of*	EIHM	[41]
*AVR-PI9*	91	7	Six cysteine	*Pi9*	BIC	[29]
*AVR-PIA*	85	5 or 7	ToxB-like	*Pia*	BIC	[17]
*AVR-PIB*	75	3	Unknown	*Pib*	BIC	[26]
*AVR-PII*	70	7	Unknown	*Pii*	BIC	[17]
*AVR-PIK/KM/KP*	113	1	ToxB-like	*Pik/Pik-m/Pik-p*, *Pik-h*	EIHM	[17]
*AVR-Pizt*	108	7	ToxB-like	*Piz-t*	BIC	[31]
*ACE1*	4035	1	PKS/NRPS	*Pi33*	EIHM	[90]
*AVR-PITA*	224	3	Zinc metalloprotease	*Pi-ta*	BIC	[51]
*AVR1-CO39*	39	1	ToxB-like	*Pi-CO39*	EIHM	[85]
*PWL1*	147	2	Glycine-rich	Unknown	BIC	[86]
*PWL2*	175	2	Glycine-rich	Unknown	BIC	[87]
*MoHTR1*	Unknown	Unknown	zinc finger TF	Unknown	BIC	[19]
*MoHTR2*	Unknown	Unknown	zinc finger TF	Unknown	BIC	[19]

Chr. No: Chromosome number; EIHM: extra-invasive hyphal membrane; BIC: biotrophic interface complex.

**Table 3:** List of None-AVR cloned effectors.

Effector Name	Description/Localization	Reference
4 biotrophy-associated secreted proteins		
Bas1	encodes a small unique protein/BIC, cytoplasm	[140]
Bas2	a small Cys-rich secreted protein/BIC, cell wall crossing points	[140]
Bas3	a small Cys-rich secreted protein//BIC, cell wall crossing points	[140]
Bas4	a small Cys-rich interfacial matrix protein/EIHM	[140]
8 secreted proteins which were necessary for pathogenicity		
Mpg1	class I fungal hydrophobin/hydrophobic surfaces	[141]
Emp1	extracellular matrix protein 1/*Cytoplasm*	[142]
Mhp1	class II fungal hydrophobin/hydrophobic surfaces	[143]
Slp1	secreted LysM Protein1/Apoplast	[144]
Mc69	encodes a hypothetical 54-amino-acid protein with a signal peptide/Apoplast	[145]
MoChia1	chitinase 1 binds to chitin/Apoplast	[18]
MoAo1	apoplastic ascorbate oxidases/Apoplast	[146]
Rbf1	a specific gene Required-for-Focal-BIC-Formation 1/BIC, cytoplasm	[147]
13 suppressors of plant cell death proteins		
Iug4/6/9	isolate unique genes/BIC; cytoplasm	[148,149]
Nup1/2/3	nucleoporins/Nuclear, cytoplasm	[149]
MoHeg13	*M. oryzae* Hypothetical Effector Genes/Apressorium	[150]
Spd2/4/7/8/9/10	suppressors of plant cell death (SPD) effectors/Apoplast and cytoplasm	[151]
12 plant cell death-inducing proteins		
MoHrip1/2	HR-inducing protein elicitor/Apoplast	[152,153]
MoCdip1 to MoCdip5	*M. oryza* cell death–inducing proteins/Apoplast	[154]
Msp1	cerato-platanin family/Apoplast	[155]
MoNlp1 to MoNlp4	nep1-like protein family of *M oryzae/*Cytoplasm	[156]
MoSm1	cerato-platanin family/Apoplast	[157]

## Data Availability

The presented data are included in the manuscript.
